# Editorial: Improving financial decisions

**DOI:** 10.3389/fpsyg.2023.1171950

**Published:** 2023-03-30

**Authors:** Irina Anderson, Volker Thoma, Daniel C. Krawczyk

**Affiliations:** ^1^School of Psychology, University of East London, London, United Kingdom; ^2^Department of Psychiatry, The University of Texas at Dallas, Richardson, TX, United States

**Keywords:** financial decision-making, cognition, social psychology, behavioral science, behavioral finance, psychology

There is a growing awareness of the importance of successful financial decision making (FDM) to individuals, organizations, and society. The ability to make financially sound decisions has been identified by prominent agencies such as The World Bank (2023) as the key enabler in reducing global poverty, boosting shared prosperity and “upskilling” people in decisional techniques that can help them to lead happier and healthier lives. The United Nations The Millennium Project (2022) considers FDM as a key issue facing humanity because of the potentially devastating individual and societal costs of being ill-equipped to make good financial decisions such as those involved when using services for credit and insurance, starting and expanding businesses, investing in education and health, managing risk, avoiding falling victim to fraud, and weathering financial shocks.

This Research Topic converges on behaviorally-focused advances in FDM with an emphasis on the interplay between individual and contextual factors that drive financial decision-making processes. The Research Topic incorporates six articles: Four are empirical investigations and two review articles of the field of FDM. The articles represent a variety of subject areas such as psychology, economics, business and applied behavioral science, and use a variety of methodological techniques including a hypothetical stock market experiment, a quasi-experimental field study, and a survey analysis of national data. Despite this diversity, a commonality of these studies lies in going beyond simple person vs. situation variables by interrogating the complexities of the interplay between these factors. Conceptualizing these interactions in their relevant contexts may give rise to a better understanding of FDM behavior and lead to concrete suggestions for interventions.

In *Mapping the knowledge domain of financial decision making: A scientometric and bibliometric study*, Guo et al. demonstrate the growing importance of a decision-focused understanding of FDM. The study reports bibliographic records collected from the Web of Science (Thomson Reuters) database for a macroscopic overview of recent research in financial decision making. The review reveals a year-on-year increase in the publication of academic articles on FDM topics. The most frequent topics of investigation across the sampling period include “Financial decisions” and “Investment decisions,” suggesting an international growth in research interest in FDM. The citation burst analysis on the data conducted by the authors predicts a future focus on individual differences such as financial literacy, gender, and age as key areas for investigation.

In their paper entitled *Peer Effects on Real-Time Search Behavior in Experimental Stock Markets*, Jin et al. examine the effect of peer decision-making on cognitive search processes for commodity pricing data in an experimental stock market task. Peer effects are important to investment decisions because the herding behavior that they engender may lead to panicked stock market performance and suboptimal FDM. Findings demonstrated a significant effect of others' decisions on pricing search behavior when selling stocks that may be detrimental to individual decision making. However, providing participants with information about how others searched for pricing options extinguished the effect of peer influence on FDM. The authors speculate that the valuable information contained in the feedback to individual decision makers gave them the confidence to conduct independent FDM searches and ignore the social influence effect of their peers' decisions. The authors conclude that FDM may be improved by provision of informational feedback to investors. Doing so may not only mitigate against potentially risky herding effects but also directly act on individual cognition.

Using a nationwide survey Kadoya et al. focus on individual and socio-economic profiles of participants who may become victims of financial fraud such as fictitious billing fraud, loan guarantee fraud, and refund fraud (*Who Is Next? A Study on Victims of Financial Fraud in Japan*). Findings showed that individual characteristics and type of fraud were important considerations in becoming victim of fraudulent incidents. For example, males, married, and financially less satisfied people were more often victims of fictitious billing fraud while older, asset-holding, and less-income-generating respondents were more likely to be victims of refund fraud. Financially less-literate people were more likely to become victims of fictitious billing fraud and loan guarantee fraud while respondents who lived with their family, those who did not have careful buying habits, and those who reported a loneliness profile were more likely to be victims of any financial fraud. These results suggest that individual characteristics, type of fraud, and the situational contexts of financially vulnerable people should all be considered in future FDM improvement strategies. In the two studies by Peetz et al. (*The Role of Income Volatility and Perceived Locus of Control in Financial Planning Decisions*) the authors observed the operation of both individual and contextual factors in FDM under conditions of uncertainty for the decision maker such as during times of income volatility. Findings showed that income volatility leads to greater personal financial insecurity and engenders decision environments that discourage planning ahead on personal finances. An internal locus of control mediated the relationship between income volatility and financial planning decisions. It also predicted financial planning decisions independently of volatility. The mediating role of this internal attributional process suggests an attempt by decision makers to exert control over what may be perceived by them to be an uncontrollable FDM environment. This research highlights not only the importance of both individual and situational factors in FDM but also the decision-maker's attempts to stabilize their FDM environments.

After an overview of more traditional approaches in FDM in *The contribution of activity theory to modeling multi-actor decision-making: A focus on human capital investments*, Marocco and Talamo's describe Activity Theory which is both a framework as well as a tool to navigate the complex nature of investment decisions in human capital projects such as startup funding provided by venture capital firms. Activity Theory proposes complex, multilayer decision-making environments allowing for dynamic interactions between different actors with often originally non-aligned objectives. The authors contend that improvements in FDM in the arena of human capital investing will need researchers and stakeholders to shift their focus from traditional psychological sites of investigation-the individual and the environment-to the more dynamic and interactional units of analysis such as, in this study, the decisional activity itself.

In *A behaviorally informed financial education program for the financially vulnerable: Design and effectiveness*, de Brujin et al. compare a traditional training programme that improves FDM skills by transferring knowledge and skill-building from educator to learner, with a novel behaviorally-informed one that aims to supplement these elements of the traditional program with additional elements that focus on improving the learner's motivation to learn about FDM and actual practice in FDM skills. The authors report robust positive effects of the behaviorally-informed program-albeit not superior to the traditional version-on financial skills, knowledge, and self-reported financial behavior. This finding suggests that an internal self-perception of success in terms of increased knowledge and skills in FDM may be a sufficient condition to improve financial decisions more generally.

In conclusion, this Research Topic provides a snapshot of a current multidisciplinary and multi-method collection of investigations into FDM with an emphasis on the need to consider the dynamic complexity of human financial decisions when investigating contributing factors (e.g., individual, group, task, goals, situation). The following aspects are provided as references for research on improving financial decisions: A focus on either individual or situational factors in FDM underestimates the complexity of processes and behavior; the consideration and use of different methods for generating knowledge about FDM processes likely provides a more differentiated understanding of how financial decisions are made and how to improve them; drawing on studies from a variety of FDM fields such as behavioral science, psychology, economics and business may offer an improved understanding of how to translate results from empirical observations into applied contexts.

## Author contributions

All authors listed have made a substantial, direct, and intellectual contribution to the work and approved it for publication.
